# Pre-exposure to phosphate-buffer saline (PBS) renders some *Pseudomonas aeruginosa* strains susceptible to triclosan on solid medium

**DOI:** 10.1093/femsle/fnag086

**Published:** 2026-07-24

**Authors:** Marine Lebrun-Corbin, Somalisa Pan, Preeti Garai, Alan R Hauser

**Affiliations:** Department of Microbiology-Immunology, Feinberg School of Medicine, Northwestern University, Chicago, IL 60611, United States; Department of Microbiology-Immunology, Feinberg School of Medicine, Northwestern University, Chicago, IL 60611, United States; Department of Microbiology-Immunology, Feinberg School of Medicine, Northwestern University, Chicago, IL 60611, United States; Department of Microbiology-Immunology, Feinberg School of Medicine, Northwestern University, Chicago, IL 60611, United States; Department of Medicine, Division of Infectious Diseases, Feinberg School of Medicine, Northwestern University, Chicago, IL 60611, United States

**Keywords:** *Pseudomonas aeruginosa*, triclosan, antibiotic resistance, selection agar, phosphate-buffered saline, resistance

## Abstract

Triclosan is an antibiotic frequently used to selectively isolate *Pseudomonas aeruginosa* from environmental and animal samples. Here, we report that while nine tested *P. aeruginosa* strains were indeed triclosan-resistant when grown on Luria Bertani agar supplemented with triclosan, all nine exhibited decreased CFU by plating following pre-exposure to phosphate-buffer saline (PBS). Compared to growth on Luria Bertani agar in the absence of PBS incubation, PBS pre-exposure was associated with mean CFU reductions ranging from ∼25% (clinical isolate PABL048) to ∼80% (laboratory strain PA14). The potential for triclosan agar to cause significant errors in experiments designed to quantify *P. aeruginosa* numbers was demonstrated using a mouse model of *P. aeruginosa* gastrointestinal carriage. In this experiment, *P. aeruginosa* carriage was underestimated by up to 20-fold when fecal samples were processed using PBS. These findings suggest that triclosan should be used with caution to quantify *P. aeruginosa* numbers in protocols that utilize PBS.

## Teaser

Following PBS exposure, the antibiotic triclosan may decrease recovery of *Pseudomonas aeruginosa*.

## Introduction

*Pseudomonas aeruginosa* thrives in a wide range of environments such as water, soil, and hospital settings (Gessard 1984, Huhulescu et al. 2011, Guida et al. 2016). The bacterium is a leading cause of nosocomial infections and can be isolated from numerous body sites, including the urinary tract, respiratory tract, bloodstream, and gastrointestinal tract (Iglewski 1996, Magill et al. 2014, Weiner et al. 2016).

Triclosan (also called irgasan) is an antibacterial agent with broad activity that is widely used in commercial products. It inhibits the enoyl-acyl carrier protein reductase FabI, thereby blocking fatty acid synthesis (McMurry et al. 1998). However, *P. aeruginosa* produces two versions of this enzyme: the typical FabI and a second enzyme, FabV, which has a low binding affinity for triclosan and allows *P. aeruginosa* to survive in the presence of this antibiotic (Zhu et al. 2009, McFarland et al. 2021). In addition, the efflux pumps of *P. aeruginosa* have been implicated in triclosan resistance (Chuanchuen et al. 2003). As a consequence, *P. aeruginosa* is viewed as being resistant to triclosan, a trait that is relatively specific to *Pseudomonas* spp. (Zhu et al. 2009, Welsch and Gillock 2011, McFarland et al. 2021). This observation has led to the frequent use of triclosan-supplemented agar plates for the selective growth of *P. aeruginosa* from polymicrobial clinical and environmental samples (Welsch and Gillock 2011, Skurnik et al. 2013, Bachta et al. 2020). Such plates are commercially available under the name “*Pseudomonas* isolation agar” (ThermoFisher Scientific 2024, Hardy Diagnostics 2024). However, it is not clear whether this resistance is a feature of all *P. aeruginosa* strains and under all conditions typically used during the isolation and processing of *P. aeruginosa-*containing samples.

Here, we demonstrate that some strains of *P. aeruginosa* yield decreased CFU on Luria Bertani (LB) agar plates supplemented with triclosan following brief exposure to phosphate-buffered saline (PBS). Because PBS is frequently used to process *P. aeruginosa* samples in clinical and laboratory settings, these findings have important implications to investigators who quantify *P. aeruginosa* numbers.

## Materials and methods

### Bacterial strains and growth conditions

PABL004, PABL006, PABL012, PABL024, PABL048, PABL049, and PABL054 are well characterized archived *P. aeruginosa* clinical isolates cultured between 1999 and 2003 from the bloodstream of patients at Northwestern Memorial Hospital in Chicago (Scheetz et al. 2009). PAO1 and PA14 are two commonly used reference strains of *P. aeruginosa* (Grace et al. 2022). Relevant characteristics of these strains are listed in Supplementary Table 1. Unless otherwise stated, bacteria were streaked onto either LB (Lennox; Sigma–Aldrich, St. Louis, MO, USA) agar plates or Vogel–Bonner minimal media (VBM) (Vogel and Bonner 1956) agar plates. When indicated, supplementation with 5 µg/ml triclosan (lot 039M4899V, Sigma–Aldrich, St. Louis, MO, USA or lot 10244018, Thermo Fisher Scientific, Waltham, MA, USA) was used.

### *Pseudomonas aeruginosa* susceptibility to triclosan in liquid medium

Bacterial cultures were grown overnight in LB medium at 37°C with shaking, then diluted 1:50 into 5 ml of fresh LB medium and incubated at 37°C with shaking for 3 h. Cultures were then centrifuged at 13 000 x *g* for 30 s, and bacterial pellets were resuspended in either LB or PBS, as indicated. The optical density at 600 nm (OD_600_) was adjusted to 0.2–0.3 using either LB or PBS, as indicated, and subsequently diluted 1:1 000. Bacterial suspensions in either LB or PBS were then diluted 1:10 in a final volume of 200 µl of LB or LB supplemented with 5 µg/ml triclosan in 96-well plates. Growth curves were generated by measuring the OD_600_ over the following 24 h using a SpectraMax iD3 instrument (Molecular Devices, San Jose, CA) set to 37°C. Throughout overall preparation of the PBS samples, it is estimated that bacteria were exposed to PBS for ∼30 min.

### *Pseudomonas aeruginosa* susceptibility to triclosan on agar plates

Bacterial cultures were grown overnight in LB medium at 37°C with shaking, then diluted 1:50 into 5 ml of fresh LB medium and incubated at 37°C with shaking for 3 h. Cultures were centrifuged at 13 000 x *g* for 30 s, and bacterial pellets were resuspended in either LB or PBS, as indicated. The OD_600_ was adjusted to 0.30–0.35 using LB or PBS, as indicated. For enumeration, the “drip-plating” method was used with LB agar, VBM agar, or LB agar supplemented with 5 µg/ml triclosan. Drip-plating was performed as follows: bacterial suspensions were serially 1:10 diluted in LB or PBS, as indicated, and 10 µl drops of each dilution were placed near the perimeter of an agar plate, the plate was tilted to allow the drops to flow across the agar surface, and the plate was incubated overnight at 37°C for subsequent CFU enumeration.

### *Pseudomonas aeruginosa* susceptibility to triclosan on *Pseudomonas* isolation agar

*Pseudomonas* isolation agar (PIA) plates containing 25 µg/ml triclosan were prepared from commercially available PIA powder (Difco 292710, Becton, Dickinson and Company, Sparks, MD, USA.) following the manufacturer’s instructions. Bacterial cultures were grown, centrifuged, and diluted as described in the preceding section. Bacterial suspensions were serially diluted in LB medium or PBS as indicated and plated onto LB agar and PIA plates.

### Quantification of fecal *P. aeruginosa* numbers in a murine model of gastrointestinal carriage

*P. aeruginosa* gastrointestinal (GI) carriage was established as previously described (Lebrun-Corbin et al. 2025). Briefly, six- to eight-week-old female C57BL/6 mice (Jackson Laboratory) received 200 µl of vancomycin (370 mg/kg, Hospira, Lake Forest, IL) daily via intraperitoneal injections for seven days. On the fifth day of vancomycin treatment, mice were orogastrically gavaged (20 G x 30 mm straight animal feeding needle, Pet Surgical, Phoenix, AZ) with 50 µl (∼10^6^ CFU) of *P. aeruginosa* resuspended in PBS as described in the preceding paragraph. To determine the level of bacterial GI carriage, feces were collected (∼10–100 mg/day) over subsequent days, weighed, homogenized in 1 ml of PBS using a BeadBlaster (Benchmark Scientific, Sayreville, NJ), and centrifuged for 30 s at 1100 x *g*. The supernatants were serially diluted in PBS and plated onto either LB agar supplemented with triclosan or VBM agar for CFU enumeration.

Mice were housed in the containment ward of the Center for Comparative Medicine at Northwestern University. This study was carried out in accordance with the recommendations in the Guide for the Care and Use of Laboratory Animals of the National Institutes of Health. The protocol was approved by the Institutional Animal Care and Use Committee at Northwestern University (Protocol Number: IS00017847). Carbon dioxide or euthasol were used as primary agents for euthanasia, and cervical dislocation was used as a secondary method.

### Statistical analysis

Unless otherwise stated, statistical analyses were performed using GraphPad Prism software (version 10.4.1). Details are provided in the figure legends.

## Results

We compared the ability of seven clinical isolates and two laboratory strains of *P. aeruginosa* to grow in liquid medium in the presence or absence of triclosan. Bacteria were grown in liquid LB with or without supplementation with 5 µg/ml triclosan. This concentration of triclosan is similar to those routinely used in research studies and is five times lower than that used in commercially available *Pseudomonas* isolation agar (ThermoFisher Scientific 2024, Hardy Diagnostics 2024). Consistent with the reported resistance of *P. aeruginosa* to triclosan, none of the strains exhibited a significant growth defect in the presence of triclosan during early- and mid-log phase, although one strain (PABL054) showed lower turbidity during late-log phase, which resulted in a statistically significant decrease in overall growth (Supplemental Fig. 1). These findings suggest that most *P. aeruginosa* strains are resistant to triclosan when grown in LB liquid medium.

**Figure 1:**
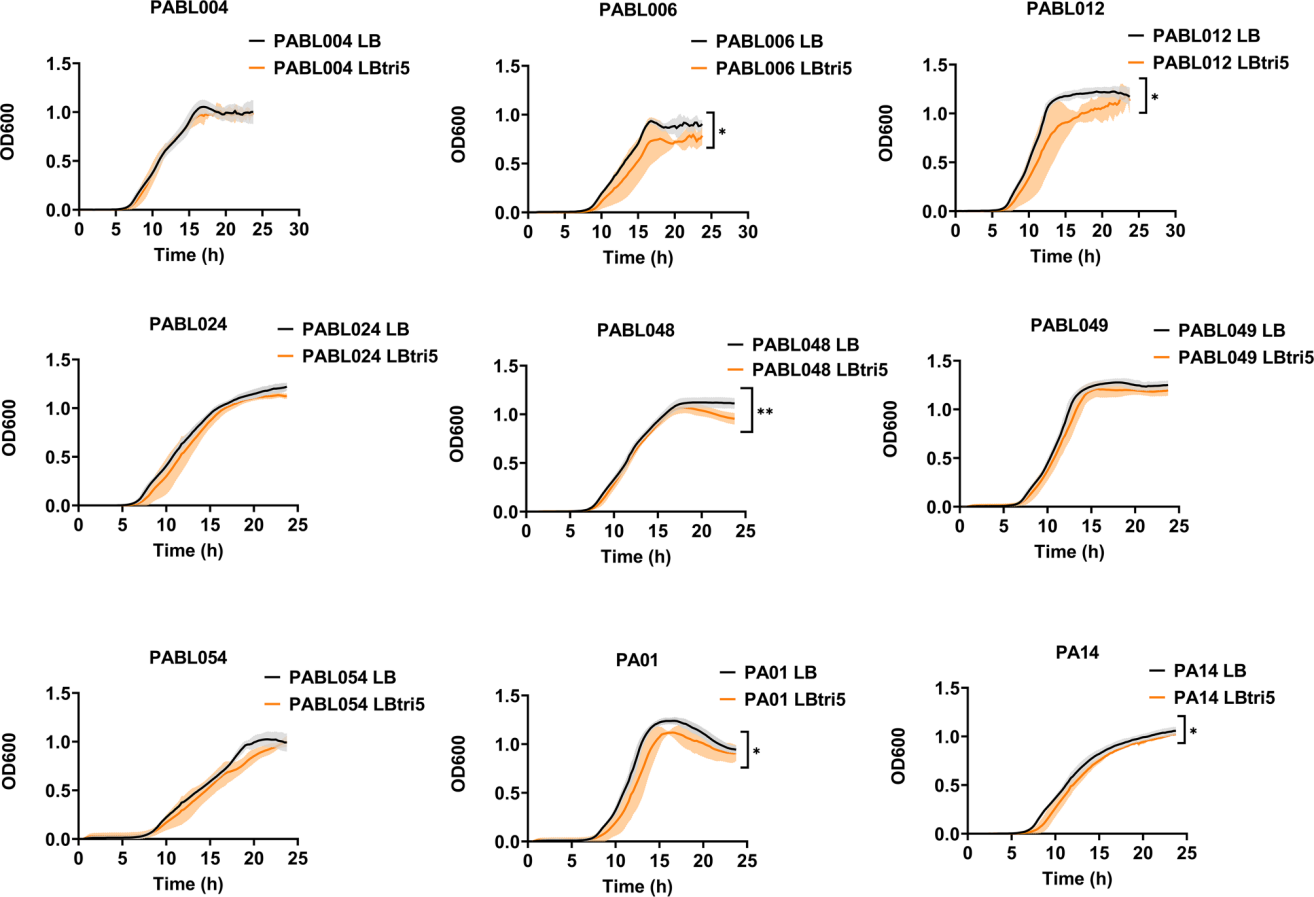
Growth curves of *P. aeruginosa* strains in liquid LB with and without triclosan supplementation following pre-exposure to PBS. Following pre-exposure to PBS, optical densities at 600 nm (OD_600_) were measured for each strain grown in LB with (orange) or without (black) 5 µg/ml triclosan. Points indicate the mean absorbance, and error bars represent standard deviations. Each experiment was performed at least three times, and results were combined. “LBtri5”, LB supplemented with 5 µg/ml triclosan. Statistically significant differences in growth are indicated by asterisks (Student’s t-test applied to area under growth curve). *P ≤ 0.05, **P ≤ 0.01.

To mimic laboratory experiments in which *P. aeruginosa-*containing samples are sometimes collected or processed in PBS (ESwab® Liquid Based Collection and Transport 2024, Puritan Medical Products Co. LLC E 2026), we repeated these experiments but used PBS in place of LB for the resuspension, OD_600_ adjustment, and dilution steps. In these experiments, it is estimated that total exposure to PBS was ∼30 min. Under these conditions, five strains (PABL006, PABL012, PABL048, PAO1, PA14) showed statistically significant triclosan-mediated growth defects following exposure to PBS (Fig. 1). Some of these strains showed slower growth during log phase. These findings suggest that exposure to PBS may cause some strains to become susceptible to triclosan when grown in liquid LB medium.

**Figure 2:**
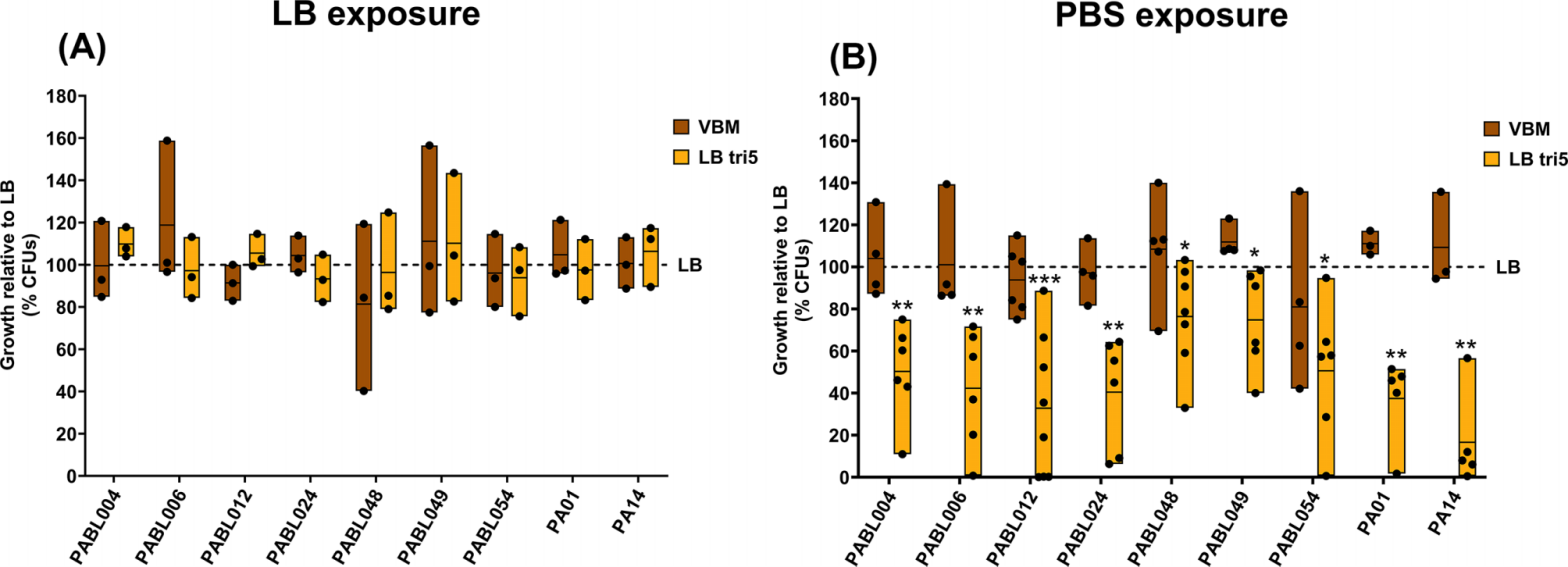
*Pseudomonas aeruginosa* CFU enumerated by plating on LB, LB with triclosan, or VBM agar. (A) Bacteria were processed in LB and plated onto LB agar with or without 5 µg/ml triclosan supplementation or onto VBM agar. (B) Bacteria were exposed to PBS during sample processing and plated onto LB agar with or without 5 µg/ml triclosan or onto VBM agar. The experiments were performed at least three times per strain and the results combined. The dashed lines indicate CFU on LB agar, which is designated 100% growth. The percentages of CFU on LB + triclosan agar or on VBM agar relative to CFU on LB agar are plotted. Filled bars represent the minimum and maximum percentages, and the horizontal lines within the bars indicate the means. The statistical significance of the percentages of CFU on LB + triclosan or VBM relative to LB agar was calculated using a one sample t-test. Without PBS exposure (A), differences between LB + triclosan and LB or VBM and LB for all strains were not significant. Following PBS exposure (B), differences between VBM and LB for all strains were not significant, whereas differences between LB + triclosan and LB are indicated as follows: *P ≤ 0.05, **P ≤ 0.01, ***P ≤ 0.001. LB-tri5, LB supplemented with 5 µg/ml triclosan.

**Figure 3:**
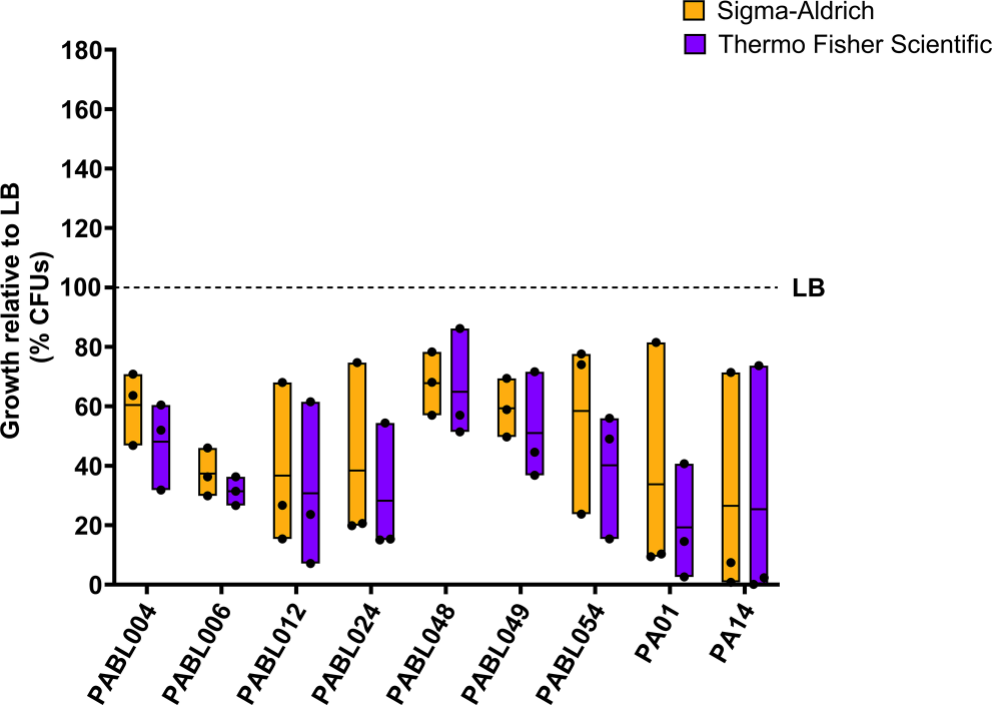
*Pseudomonas aeruginosa* CFU counts on LB agar plates supplemented with triclosan from two different vendors. Bacteria were processed in PBS and plated onto LB agar with or without supplementation (5 µg/ml) with triclosan from Sigma–Aldrich (orange) or Thermo Fisher Scientific (purple). The experiment was performed three times per strain, and the results were combined. The dashed line indicates CFU on LB agar, which is designated 100% growth. The percentages of CFU on LB + triclosan agar relative to CFU on LB agar are plotted. Filled bars represent the minimum and maximum percentages, and the horizontal lines within the bars indicate the means. No statistically significant CFU differences were observed between plates using triclosan from Sigma–Aldrich and triclosan from Thermo Fisher Scientific (multiple unpaired t-tests).

To better quantify the effects of triclosan on PA recovery following exposure to PBS and to more closely mimic experimental protocols that utilize PBS, we assessed growth on agar plates instead of liquid medium. We first grew *P. aeruginosa* in liquid LB, centrifuged the culture, resuspended in LB and plated onto LB agar with or without 5 µg/ml triclosan supplementation. Plates were incubated overnight at 37°C, and CFU were enumerated the following day. None of the strains exhibited a significant defect in CFU formation in the presence of triclosan (Fig. 2A). We then repeated these experiments using PBS in place of LB for resuspension, OD_600_ adjustment, and dilution steps, resulting in exposure to PBS for ∼30 min. The bacteria were then plated onto LB agar with or without 5 µg/ml triclosan supplementation (Supplemental Fig. 2). All nine strains showed a statistically significant decrease in CFU on the agar plates containing triclosan compared to the agar plates lacking triclosan (Fig. 2B). The differences ranged from a ∼25% (clinical isolate PABL048) to ∼80% (laboratory strain PA14) reduction in mean CFU. Similar results were obtained using triclosan purchased from two different vendors (Fig. 3). These findings indicate that pre-exposure to PBS caused decreased recovery of CFU following plating onto triclosan-supplemented LB agar.

VBM medium has also been used to select for growth of *P. aeruginosa* (Vogel and Bonner 1956, Hmelo et al. 2015, Greene et al. 2018, Schinner et al. 2020). Since VBM selectivity is based on the metabolic versatility of *P. aeruginosa* rather than resistance to triclosan, we hypothesized that VBM would not impair CFU recovery. To test this, bacteria were grown and processed as described in the preceding paragraphs (including exposure to PBS) before being plated on VBM agar. Total CFU recovery on VBM was comparable to that observed on LB agar plates even following exposure to PBS (Fig. 2B). These findings indicate that VBM agar plates are suitable for recovering and quantifying *P. aeruginosa* from samples pre-exposed to PBS and that PBS exposure alone is not sufficient to reduce recovered *P. aeruginosa* numbers.

**Figure 4:**
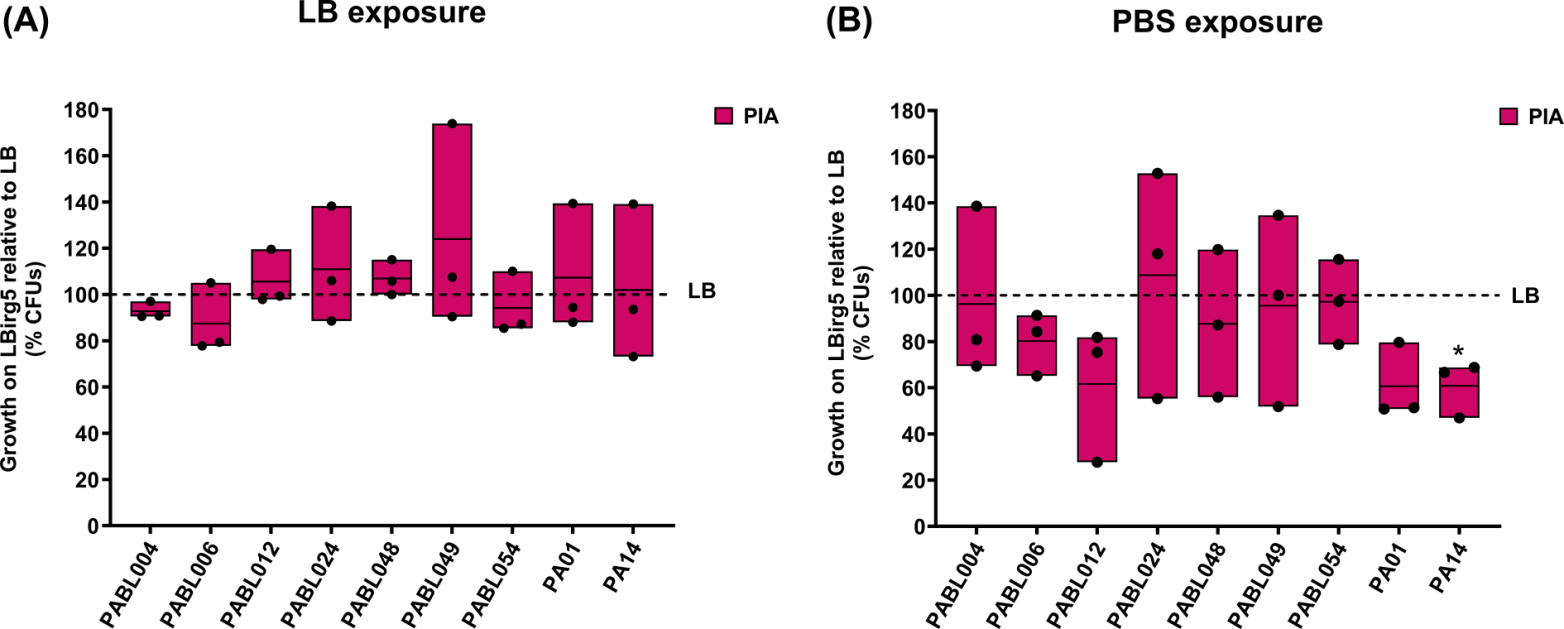
*Pseudomonas aeruginosa* CFU counts on LB agar and *Pseudomonas* Isolation Agar (PIA). Bacteria were processed without (A) or with (B) PBS exposure and enumerated on LB agar plates or PIA plates. The experiments were performed at least three times per strain, and the results combined. The dashed lines indicate CFU on LB agar, which is designated 100% growth. The percentages of CFU on PIA compared to LB agar are plotted. Filled bars represent the minimum and maximum percentages, and the horizontal lines within the bars indicate the means. The statistical significance of the percentages of CFU on PIA relative to LB agar was calculated using a one sample t-test. Except for PA14, none of the differences were statistically significant (multiple unpaired t-tests), although trends towards fewer CFU on PIA following PBS exposure were noted for PABL006 (*P* = 0.13), PABL012 (*P* = 0.15), and PAO1 (*P* = 0.053). *P < 0.05.

We next determined whether pre-exposure to PBS could cause erroneous quantification of *P. aeruginosa* with the use of commercially purchased PIA. The nine strains were grown and exposed to LB medium or PBS and then plated for enumeration onto LB agar or PIA plates. As expected, the numbers of CFU obtained following exposure to LB were not statistically different on LB agar vs. PIA (Fig. 4A). Following pre-exposure to PBS, CFU recovery on PIA was significantly less than on LB agar for one strain, PA14 (*P* = 0.03) and trended towards less for three other strains, PABL006, PABL012, and PAO1 (*P* = 0.13, 0.15, and 0.053, respectively). These findings suggest that the growth inhibitory effects of triclosan following PBS pre-exposure are not as marked on PIA as on triclosan-supplemented LB agar, perhaps because of other chemical differences between these two media.

**Figure 5:**
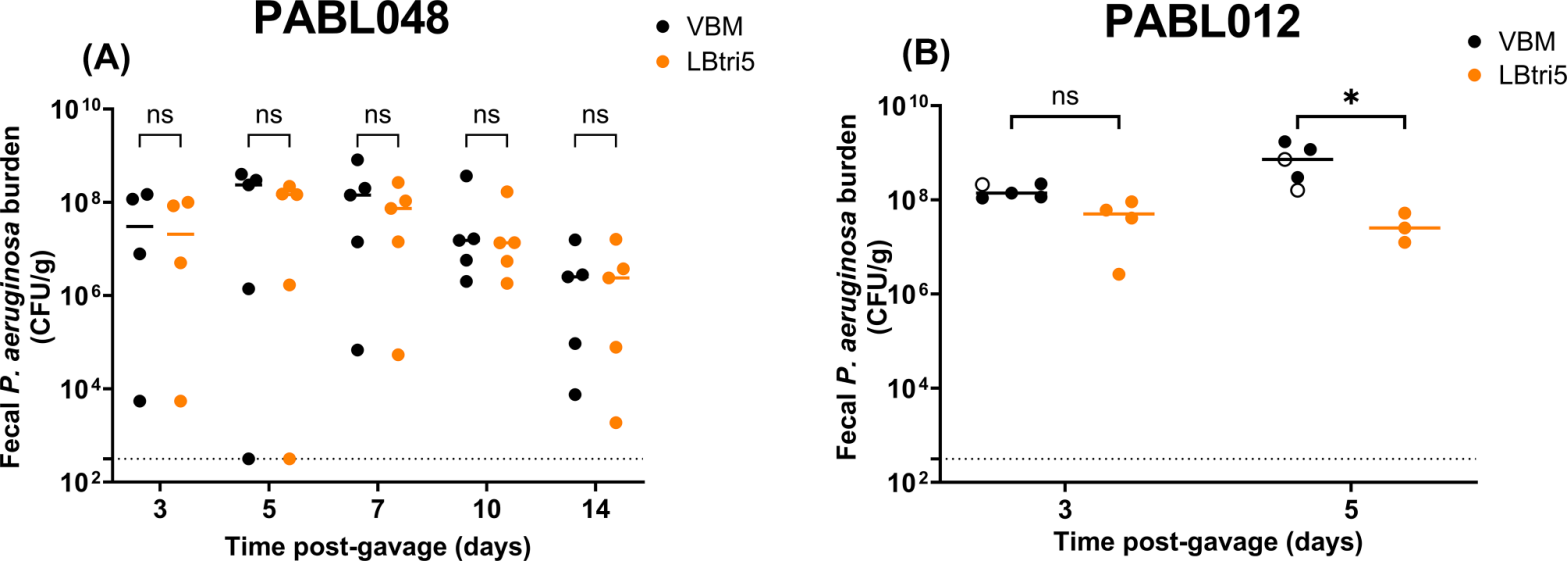
Recovery of *P. aeruginosa* CFU from mouse feces using triclosan-supplemented LB or VBM agar. Mice received vancomycin injections and an orogastric gavage with (A) 10^5.5^ CFU of strain PABL048 or (B) 10^5.8^ CFU of strain PABL012. Mouse feces were subsequently collected over the next week, homogenized in PBS, serially diluted in PBS, and applied to VBM agar (black) or LB agar supplemented with 5 µg/ml triclosan (orange) for enumeration. The experiment was performed once with 5 mice per condition. Each symbol represents one mouse. Open circles in the VBM groups indicate mice from which CFU could not be inferred from the corresponding LB-triclosan agar (i.e. when confluent growth occurred with one dilution, but no colonies were noted with the corresponding 1:10 dilution; see text and (Fig. 6). Solid horizontal bars indicate medians. Dotted lines indicate the limits of detection. Statistical differences between the VBM and LB-triclosan groups were calculated with a paired t-test. “ns”, non-significant; *P ≤ 0.05. “LBtri5”, LB supplemented with 5 µg/ml triclosan.

Finally, we tested the extent to which PBS-induced susceptibility of *P. aeruginosa* to triclosan could cause misleading results in an actual experiment. For this purpose, we used a murine model of GI carriage in which the level of *P. aeruginosa* carriage is quantified by CFU enumeration from feces (Lebrun-Corbin et al. 2025). Fecal samples were collected, homogenized in PBS, and plated on VBM and LB agar supplemented with 5 µg/ml triclosan to suppress growth of fecal bacteria other than *P. aeruginosa*. PABL048, a strain that is minimally sensitized to triclosan by PBS pre-exposure (∼25% reduction in CFU; Fig. 2B), yielded comparable results on VBM and LB-triclosan agar (Fig. 5A). In contrast, carriage of PABL012, a strain that was more sensitive to triclosan following PBS pre-exposure (∼80% reduction in CFU; Fig. 2B), was underestimated by up to ∼20-fold when LB-triclosan agar plates were used for CFU enumeration instead of VBM agar plates (Fig. 5B). We conclude that the use of triclosan to select for *P. aeruginosa* growth can lead to erroneous results in experiments that involve PBS during the processing of samples.

**Figure 6:**
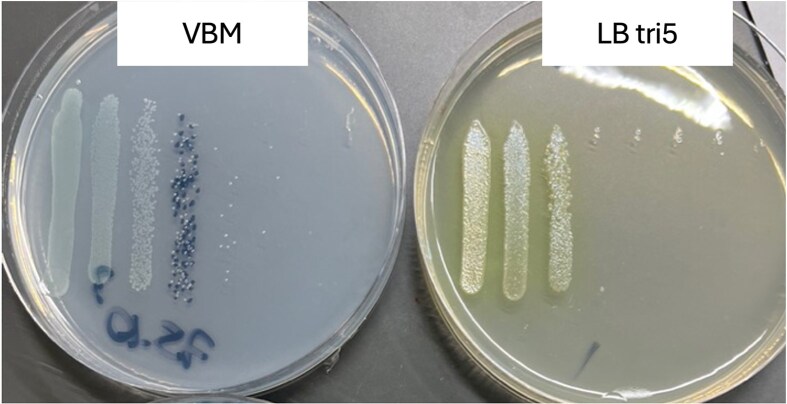
Example from mouse feces showing density-dependent CFU reduction of *P. aeruginosa* grown on triclosan-supplemented LB agar. Mice were inoculated with strain PABL012, and feces were subsequently collected, homogenized in PBS, serially diluted in PBS, and plated. Examples are shown of a VBM agar plate (left) and an LB-triclosan agar plate (right) following overnight incubation. The black dots underlying the fourth drip on the VBM agar plate were made by a marker to aid in the enumeration of CFU. “LBtri5”, LB supplemented with 5 µg/ml triclosan.

While performing the mouse experiments, we noticed another interesting feature of *P. aeruginosa* growth on triclosan-supplemented agar following incubation with PBS. On some agar plates, fecal CFU counts of PABL012 showed inconsistent results across dilutions. For some samples, bacterial suspensions yielded confluent colonies, while the corresponding 1:10 dilution of the same suspension yielded very few or no detectable colonies (Fig. 6). This phenomenon was not observed with VBM agar plates (Fig. 6) but was occasionally observed with several strains of PBS-exposed bacteria in the *in vitro* experiments described in Fig. 2, as shown for PABL012 in Supplemental Fig. 2. These findings suggest that under certain conditions the effects of triclosan on *P. aeruginosa* CFU are density-dependent.

## Discussion

Triclosan supplementation is commonly used in research and clinical settings to select for *P. aeruginosa* growth from polymicrobial samples. Our study suggests that this approach should be used with caution when the samples have been previously exposed to PBS. We found that all nine tested clinical and laboratory strains of *P. aeruginosa* displayed significantly decreased CFU on triclosan-supplemented agar plates following pre-exposure to PBS. For some strains, this reduction was substantial (e.g. ∼80% fewer CFU for strain PA14). *Pseudomonas aeruginosa* CFU were not reduced by PBS exposure alone, as shown by the normal growth of similarly pre-exposed samples on LB and VBM agar plates. However, PBS exposure may alter the stress response, membrane structure and permeability, expression of FabV or efflux pumps, or the underlying metabolic state of *P. aeruginosa*, which in turn may enhance susceptibility to triclosan. Alternatively, triclosan exposure may predispose to enhanced bacterial aggregation that in turn causes decreased CFU. In either case, the underlying mechanism appears to vary in penetrance, as some *P. aeruginosa* strains were more affected than others. We hypothesize that this phenomenon is not exclusive to PBS. Future studies should determine whether similar effects occur with other non-nutritive solutions or media containing high salt or phosphate concentrations, such as storage media used for clinical swabs and laboratory minimal media (e.g. MOPS [3-(N-Morpholino) Propane-Sulfonic Acid], M9, or MINS).

In conclusion, our findings demonstrate that *P. aeruginosa* CFU recovery on triclosan-LB agar is attenuated following exposure to PBS. Hence, investigators should be cautious when using triclosan to select for or quantify *P. aeruginosa* in procedures that involve PBS.

## Supplementary Material

fnag086_Supplemental_Files
