# Supplementary material for: Pre-exposure to phosphate-buffer saline (PBS) renders some Pseudomonas aeruginosa strains susceptible to triclosan on solid medium
Source: FEMS Microbiol Lett. 2026 Jul 24;373:fnag086. doi: 10.1093/femsle/fnag086 (PMC13436967; doi:10.1093/femsle/fnag086)
Supplement: Supplementary file 1 [file fnag086_supplemental_files.zip › Supplemental Figures with final edits.docx]

**SUPPLEMENTAL FIGURES**

**Supplemental Figure 1: Growth curves of *P. aeruginosa* strains in liquid LB medium with and without triclosan supplementation.** Optical densities at 600 nm (OD_600_) were measured for each strain grown in LB with (orange) or without (black) 5 µg/mL triclosan. Points indicate the mean absorbance of two biological replicates, and shaded areas represent standard deviations. “LBtri5”, LB supplemented with 5 µg/mL triclosan. Statistically significant differences in growth between LB and LB + triclosan are indicated by an asterisk (Student’s t-test but with only 2 replicates). *, p < 0.05.

**Supplemental Figure 2: Example of the enumeration of *P. aeruginosa* suspension CFU following exposure to PBS by plating onto LB agar with and without triclosan supplementation.** (A) Schematic representation of the drip plating technique. Each drip is 10 µL of bacterial suspension and is the result of a 1/10 dilution of the suspension used for the drip to its left. This panel was created using Biorender.com. (B) Following exposure to PBS, strain PABL012 was drip plated onto LB agar with and without triclosan supplementation (5 µg/mL). “LBtri5”, LB supplemented with 5 µg/mL triclosan.
